# Hypoxia-Inducible Factors and Burn-Associated Acute Kidney Injury—A New Paradigm?

**DOI:** 10.3390/ijms23052470

**Published:** 2022-02-23

**Authors:** Dan Mircea Enescu, Sorin Viorel Parasca, Silviu Constantin Badoiu, Daniela Miricescu, Alexandra Ripszky Totan, Iulia-Ioana Stanescu-Spinu, Maria Greabu, Viorel Jinga

**Affiliations:** 1 Department of Plastic Reconstructive Surgery and Burns, Grigore Alexandrescu Clinical Emergency Hospital for Children, Faculty of Medicine, Carol Davila University of Medicine and Pharmacy, 020021 Bucharest, Romania; dan.enescu@umfcd.ro; 2 Department of Plastic and Reconstructive Surgery, Emergency Clinical Hospital for Plastic, Reconstructive Surgery and Burns, Faculty of Medicine, Carol Davila University of Medicine and Pharmacy, 020021 Bucharest, Romania; 3 Department of Anatomy and Embriology, Faculty of Medicine, Carol Davila University of Medicine and Pharmacy, 050474 Bucharest, Romania; 4 Department of Plastic and Reconstructive Surgery, Life Memorial Hospital, 010719 Bucharest, Romania; 5 Department of Biochemistry, Faculty of Dental Medicine, Carol Davila University of Medicine and Pharmacy, 050474 Bucharest, Romania; daniela.miricescu@umfcd.ro (D.M.); alexandra.totan@umfcd.ro (A.R.T.); iulia.stanescu@umfcd.ro (I.-I.S.-S.); maria.greabu@umfcd.ro (M.G.); 6 Department of Urology, “Prof. Dr. Th. Burghele” Clinical Hospital, Faculty of Medicine, Carol Davila University of Medicine and Pharmacy, 020021 Bucharest, Romania; viorel.jinga@umfcd.ro

**Keywords:** hypoxia, hypoxia-inducible factors, burns, acute kidney injury, oxidative stress

## Abstract

O_2_ deprivation induces stress in living cells linked to free-radical accumulation and oxidative stress (OS) development. Hypoxia is established when the overall oxygen pressure is less than 40 mmHg in cells or tissues. However, tissues and cells have different degrees of hypoxia. Hypoxia or low O_2_ tension may be present in both physiological (during embryonic development) and pathological circumstances (ischemia, wound healing, and cancer). Meanwhile, the kidneys are major energy-consuming organs, being second only to the heart, with an increased mitochondrial content and O_2_ consumption. Furthermore, hypoxia-inducible factors (HIFs) are the key players that orchestrate the mammalian response to hypoxia. HIFs adapt cells to low oxygen concentrations by regulating transcriptional programs involved in erythropoiesis, angiogenesis, and metabolism. On the other hand, one of the life-threatening complications of severe burns is acute kidney injury (AKI). The dreaded functional consequence of AKI is an acute decline in renal function. Taking all these aspects into consideration, the aim of this review is to describe the role and underline the importance of HIFs in the development of AKI in patients with severe burns, because kidney hypoxia is constant in the presence of severe burns, and HIFs are major players in the adaptative response of all tissues to hypoxia.

## 1. Introduction

In the second half of 19th century, Paul Bert was the pioneer who identified hypoxemic hypoxia as the cause of altitude sickness. Since then, a huge number of experimental studies regarding hypoxia, including animal studies (rabbits), have been performed. Moreover, in 2019, the Nobel Prize was won by a group of researchers who explained how cells detect and adapt to different O_2_ concentrations [1].

It is well known that aerobic organisms produce energy in the presence of O_2_. Furthermore, in aerobic organisms, O_2_ regulates various processes involved in their development, in the response to tissue damage, in infection, and in neoplastic growth [2]. On the other hand, O_2_ deprivation induces stress in living cells, linked with free-radical accumulation and oxidative stress (OS) development [3]. Hypoxia is established when the overall oxygen pressure is less than 40 mmHg in cells or tissues [4]. However, tissues and cells have different degrees of hypoxia [5]. Hypoxia or low O_2_ tension may be present in both physiological (during embryonic development) and pathological circumstances (ischemia, wound healing, and cancer) [6].

Meanwhile, the kidneys are major energy-consuming organs, being second only to the heart, with an increased mitochondrial content and O_2_ consumption [7]. The kidneys receive O_2_ through the blood flow, with around 20–25% of the cardiac output being sent to these organs [8]. Renal tubular epithelial cells consume O_2_ and produce ATP, further used for water and solutes reabsorption from pre-urine [8]. The renal vascular architecture participates in oxygen/nutrient delivery and filtration of the blood [9]. The renal function may undergo autoregulation, and renal vessels are formed by angiogenesis and vasculogenesis [10].

Furthermore, hypoxia-inducible factors (HIFs) are the key players that orchestrate the mammalian response to hypoxia [11,12]. HIFs are transcription factors with basic helix–loop–helix DNA binding motifs, of the family of period circadian protein-aryl hydrocarbon receptor nuclear translocators-single-minded proteins (PER-ARNT-SIM) (bHLH-PAS) [12]. In mammals, the *HIF1A*—endothelial pas domain-containing protein1*EPAS1*—or *HIF2A* and *HIF3A* genes encode the α subunits, while aryl hydrocarbon receptor nuclear translocators-1 *(ARNT1)* and aryl hydrocarbon receptor nuclear translocators-2 *(ARNT2)* encode the HIF-1β subunits (*HIF1B*) [12]. HIFs activate the hypoxia signaling pathway, which further induces gene expression for processes such as angiogenesis, metabolism, and coagulation [13]. HIFs adapt cells to low oxygen concentrations by regulating transcriptional programs involved in erythropoiesis, angiogenesis, and metabolism [14]. This suggests that these programs promote the growth and progression of various types of tumors. For this reason, HIFs are important anticancer targets [14]. Phosphoglycerate kinase-1 (PGK-1), glucose transporter-1 (GLUT-1), vascular endothelial growth factor (VEGF), and erythropoietin (EPO) are HIF target genes [15].

On the other hand, one of the life-threatening complications of severe burns is acute kidney injury (AKI) [16]. By severe burns, we generally mean burns that involve more than 20% of the Total Body Surface Area (TBSA) [16]. Such burns have a high risk of producing shock [17], which is constantly associated with a more or less severe form of AKI. The reported incidence of AKI in burned patients admitted to the Intensive Care Unit (ICU) is 30–46% [18,19,20,21]. The dreaded functional consequence of AKI is an acute decline of the renal function [22], namely, Acute Renal Failure (ARF), with a reported incidence up to 30% among patients with burns [20] and a mortality between 54% and 100% [20,23,24]. Before 1965, no patients could survive ARF caused by burns [20,25].

Taking all these aspects into consideration, the aim of this review is to describe the role and underline the importance of HIFs in the development of AKI in patients with severe burns, because hypoxia of the kidneys is constant in the presence of severe burns, and HIFs are major players in the adaptative response of all tissues to hypoxia.

## 2. Hypoxia-Inducible Factors: Structure, Roles, and Involvement in Pathology

HIFs contain two subunits, the oxygen-dependent α subunit HIF-alpha (α) and the oxygen-insensitive β subunit (β) [26]. While the α subunit is expressed in hypoxic conditions, the β subunit undergoes heterodimerization in the nucleus with the α subunit and will act as a transcription factor [27]. The association of α and β subunits will enhance the expression of genes involved in glycolysis, angiogenesis, and cell survival [27].

All α subunits have a similar domain structure and are highly conserved at the protein level. Furthermore, α subunits can heterodimerize with HIF-1β and may bind to a DNA sequence named hypoxia-responsive element (HREs). This DNA binding may explain their differential influence on the expression of some genes [28].

HIF-α has three isoforms: HIF-1α, HIF-2α, HIF-3α [27]. Among all HIF proteins, the most studied regarding their activity and function are HIF-1α, HIF-2α, and HIF-1β [29]. Of the three isoforms, HIF-1α and HIF-2α are the most expressed in hypoxic conditions [30]. HIF-1α is widely distributed in all human normal and hypoxic conditions, while HIF-2α has been detected only in physiologically hypoxic tissues [30]. Moreover, HIF-2α is highly expressed in the endothelium and in interstitial cells of the kidneys [5], thus modulating vascular endothelial cell function [31]. However, Eckardt KU and his research team detected HIF-1α mainly in tubular cells, while HIF-2α was shown in peritubular interstitial, endothelial, and glomerular cells [32]. HIF-3α is found in adult thymus, lung, heart, brain, and kidney [30]. Moreover, HIF-3α may be expressed in highly vascularized tissues such as the cornea [33].

In addition, the protein levels of all HIF-α isoforms (HIF-1α, HIF-2α, and HIF-3α) are regulated by their conditional interaction with the von Hippel–Lindau tumor suppressor protein (pVHL) [34]. pVHL acts as an E3 ubiquitin ligase that targets the HIF-α-minimal N-terminal transactivation domain (N-TAD) within the oxygen-dependent degradation domain (ODD) [34].

In the presence of O_2_, prolyl hydroxylases (PHDs) catalyze the hydroxylation of HIF-1/2α at specific proline residues [35]. Further, hydroxylated HIF-1/2α is recognized by the pVHL ubiquitin ligase complex that induces its conjugation with ubiquitin and leads to its proteasomal degradation [35]. In the absence of O_2_, with the help of coactivator p300/CREB-binding protein (CBP), HIF-1/2α is stabilized and undergoes dimerization with HIF-1β, which will lead to the transcription of genes linked to hypoxia-responsive elements [35]. There are three PHD proteins involved in HIF hydroxylation: PHD1, also known as α-ketoglutarate-dependent hydroxylase-2 (EGLN2) and HIF-prolyl-hydroxylase-3 (HPH3), PHD2 (EGLN1/HPH2), and PHD3 (EGLN3/HPH1). PHD are Fe (II)- and 2-oxoglutarate (2OG)-dependent [36].

**Figure 1:**
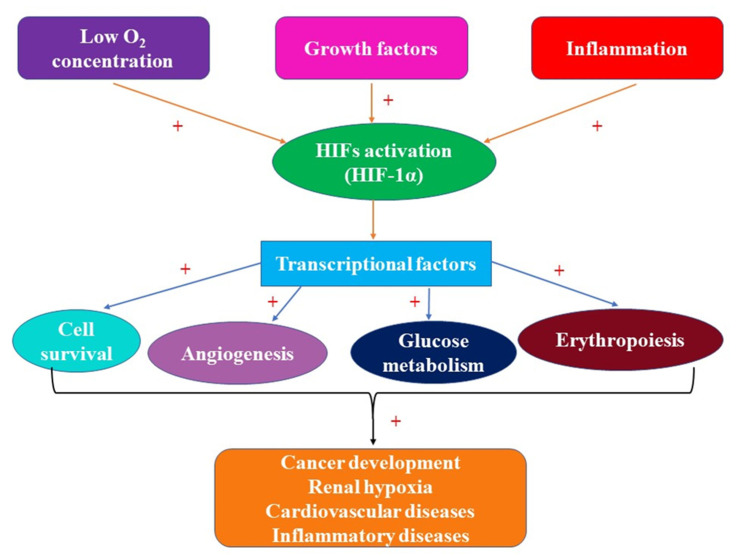
HIFs activation and involvement in pathological processes.

On the other hand, HIFs are activated not only by hypoxia (Figure 1) but also by genetic mutations related to a wide variety of tumors [37]. Besides hypoxia, HIFs expression may be induced by loss of tumor suppressors, such as pVHL, phosphatase-and-tensin-homologue (PTEN), tuberous sclerosis complex ½ (TSC1/2), progressive multifocal leukoencephalopathy (PML), and succinate dehydrogenase (SDH), and by the increased activity of the signaling pathways associated with phosphatidylinositol 3-kinase (PI3K) and Mitogen-Activated Protein Kinase (MAPK) [38]. The transcriptional activity of HIFs is influenced by posttranslational modifications including hydroxylation, acetylation, phosphorylation, and S-nitrosylation [39]. Tumor cells live in a hypoxic medium, hypoxia being correlated with tumor aggressiveness. In the presence of decreased amounts of oxygen, HIFs act as transcription factors, adapting the cells to the hypoxic conditions via glucose metabolism [40]. During cancer progression, besides its implication in glucose metabolism, hypoxia is involved in inflammation, anti-apoptosis processes, and angiogenesis [27].

Moreover, studies performed in cell cultures have shown that HIFs could be activated under normal O2 concentration by cytokines and growth factors [41]. Therefore, it is possible that under clinical conditions, associated with inflammation, infection, and sepsis, HIFs could be activated (Figure 1) [41]. This may suggest that in humans, growth factors and cytokines are preconditional activators of HIFs [41]. Thus, during infection and inflammation, the immune cell population together with O_2_ is involved in HIF-α stabilization, induced partially by the hypoxic tissue context of disease [42]. Hypoxia plays an important role in the pathology of inflammatory diseases such as chronic inflammatory bowel disease, which is a risk factor for colorectal cancer development [43]. Therefore, chronic inflammation is characterized by hypoxia [13]. Inflamed and injured tissues are characterized by increased amounts of inflammatory cytokines, reactive oxygen species (ROS), and nitrogen species, while O_2_ and glucose levels are decreased [44].

Under normoxic conditions, in the cells of the innate and adaptive immune system, HIF-1 is upregulated by bacterial and viral compounds, preparing the cells to migrate and to function in inflamed and hypoxic tissues [44]. Moreover, the activity of these cells is further enhanced by proinflammatory cytokine release [interleukine-1β (IL-1β,) tumor necrosis factor-α (TNF-α)] induced by HIF-1 [44]. In renal cancer carcinoma, HIF-1α and HIF-2α have opposite effects, HIF-1α being a tumor suppressor, and HIF-2α acting as an oncogene [45]. Moreover, this HIF-1α/HIF-2α shift promotes proinflammatory and profibrotic activities in glomerular and renal tubular cells [46]. Renal tubular epithelial cells possess all PHDs (PHD1, PHD2, and PHD3) [46].

In the core of advanced atherosclerotic plaques, a hypoxic state is established, which is correlated with neovascularization and inflammatory processes [47]. This molecular event contributes to atherosclerotic plaque instability [47], a very important aspect, taking into consideration that cardiovascular diseases are the most common cause of death worldwide [48,49,50]. Additionally, patients diagnosed with type 2 diabetes have renal hypoxia, OS, endoplasmic stress, and a nutritional deficiency that will cause HIF-1α activation and HIF-2α suppression. Studies performed so far reported that high expression levels of HIFs are correlated with poor prognosis for various cancer types [4,51,52,53,54]. Pancreatic beta cells can be subjected to O_2_ deprivation, so hypoxia can contribute to beta cells damage [55].

In addition, HIF-1α, together with IL-6, vascular endothelial growth factor (VEGF), microRNA-150, microRNA-270, ROS, bone morphogenetic protein 6 (BMP6), triggering receptor expressed on myeloid cells 1 (TREM-1), and PI3K/protein kinase B (AKT) signaling pathway, is involved in psoriasis pathogenesis [56]. It is well known that in the pathogenesis of psoriasis, cytokines such as IL-1, IL-4, IL-6, IL-8, IL-12, and TNF are involved [57].

## 3. HIFs and Acute Kidney Injury (AKI)

### 3.1. Renal Biology and AKI

Like the heart and the brain, the kidney is highly dependent on ATP availability; therefore, lack of ATP and mitochondrial dysfunction play key roles in organ dysfunction [58]. Peritubular capillary plexuses deliver O_2_ in normal kidneys, where plexuses are supplied by efferent arterioles and drained by renal venules [59]. Due to blood vessels antiparallel arrangement, the renal circulation in the presence of extensive arteriovenous malformations is shunted [59]. This decreases renal tissue O_2_ extraction [59]. Although the kidneys receive a large amount of blood perfusion, with respect to their weight, the renal cortex and the inner medulla have a decreased O_2_ tension [59]. Kidneys are also prone to aging, characterized by progressive tubular dysfunction and decreased sodium reabsorption, potassium excretion, and urine concentrating capacity, which will increase AKI development [60,61]. In the pathogenesis of age-related kidney damage, various processes are involved, including OS, inflammation, mitochondrial dysfunction, altered calcium regulation, and RAS activation [62].

AKI was defined and staged by several criteria systems (scoring systems), in relation to serum creatinine levels with or without urine output [63].

The RIFLE criteria (Risk, Injury, Failure; Loss, End-Stage Renal Disease criteria) are based on serum creatinine levels, glomerular filtration rate, and urine output. AKI is defined by an increase in serum creatinine level of more than 50% above the baseline developing over less than 7 days; urine output should be less than 0.5 mg/kg/h for a minimum of 6 h. According to the RIFLE criteria there are five stages of severity: Risk, Injury, Failure, Loss, End stage [64].

The AKIN criteria (Acute Kidney Injury Network criteria) are based on serum creatinine levels and urine output. AKI is defined by an increase of serum creatinine level of 0.3 mg/dL or an increase of serum creatinine level of more than 50% in less than 48 h. Urine output is less than 0.5 mg/kg/h for a minimum of 6 h. According to the AKIN criteria, there are three stages of severity: Risk, Injury, Failure [65].

The KDIGO criteria (Kidney Disease Improving Global Outcomes criteria) are based on serum creatinine levels and urine output. AKI is defined by an increase of serum creatinine level of 0.3 mg/dL developing over 48 h or an increase of serum creatinine level of more than 50% developing over 7 days. Urine output is than 0.5 mg/kg/h for a minimum of 6 h. According to the KDIGO criteria, there are three stages of severity: Risk, Injury, Failure [63].

The scoring systems presented above appeared to provide similar results when applied to large cohorts of patients (at least for AKI in burn patients) [65,66,67].

There are two types of burn-associated acute kidney injuries: early post-burn and late post-burn [20]. Early post-burn-associated AKI develops in the first 48–72 h from the initial injury. The etiology is multifactorial: burn shock and hypovolemia, under-resuscitation, over-resuscitation, cardiac dysfunction, denatured proteins, rhabdomyolysis, inflammatory mediators [20,68]. Late post-burn-associated AKI develops after 3–4 days from the initial burn injury. The etiology includes sepsis, nephrotoxic drugs, over-resuscitation, multiorgan failure [20,68].

### 3.2. HIFs and AKI

In most cases, the common denominator of acute kidney injury is tissue hypoxia, which is the canonic activator of HIFs [69,70] in the kidneys and in other organs (brain, liver, heart, lungs) and tissues (smooth muscles, endothelium). The HIF signaling pathway might be also activated by non-canonical activators, such as TNF-α [71], interleukin-1β (IL-1β) [72,73], insulin [73,74], insulin-like growth factors [74,75,76], angiotensin II [77], nitric oxide [78,79,80], and ROS [81,82,83].

Why is HIFs activation important? Because, as transcriptional factors, HIFs regulate (directly or indirectly) the expression of genes that facilitate oxygen delivery to the renal (and other) tissues and promote adaptation to hypoxia in the kidney (and other organs—brain, liver, heart, lungs) [84,85]. Oxygen delivery is improved through angiogenesis and augmentation of the oxygen transport capacity of the blood (via erythropoiesis) [86]. Oxygen utilization by the cells is made more efficient through a shift of the metabolism towards glycolysis [87]. HIFs literally upregulate the genes encoding most of the glycolytic enzymes [86,88].

The HIF target genes are also involved in iron metabolism, cell proliferation, apoptosis, intercellular interaction, matrix–cells interaction, adenosine metabolism, NO (nitric oxide) metabolism [84,85], redox homeostasis, autophagy, immune response [87,88,89,90]. The HIF target genes direct the synthesis of EPO, VEGF, PGK-1, GLUT-1, transferrin and transferrin receptor, enolase 1, lactate dehydrogenase A(LDH-A), CTGF (connective tissue growth factor), and many other molecules [84], being essential for kidney functionality in normal conditions and for kidney adaptation to hypoxia [15,91]. It is well known that HIF target genes have regulatory regions containing identifiable hypoxia response elements (HREs). There are over 200 HIF target genes, not all of them being regulated by an HRE [84]. A completely functional HRE contains an HBS (HIF DNA-binding site) and several neighboring DNA-binding sites (for other transcription factors than HIF) [84,90]; these are binding sites with sequence motifs for stress-responsive transcription factors, not necessarily hypoxia-inducible: AP-1 (activator protein 1), CREB (cAMP response element-binding), CEBP (CCAAT-enhancer binding protein) [92]. They are supposed to augment the response to hypoxia and confer HRE tissue-specificity [84]. Therefore, there is a cooperation between HIFs and other transcription factors, for example, for the expression of the LDH-A gene, where HIF1 cooperates with ATF-1/CREB-1 transcription factor [92,93], and for the VEGF gene, where HIF1 cooperates with AP-1 binding factors [84]. There is a 20-fold increased binding of HIF1 and HIF2 to normoxic DNAse1 hypersensitivity sites [92,94] and there is an enhanced HIF1 binding to DNA sequences close to genes with normoxia non-restrictive transcriptional state [92,95]. This suggests that the selective access of transcription factors to binding sites is partially conditioned by DNA regional and conformational accessibility in normoxic conditions [96,97]. Hence, the specific tissue effects of HIFs.

### 3.3. Severe Burns, AKI, and HIFs

In patients with severe burns, there is a biphasic metabolic response [98]. First, the “Ebb” Phase, which develops immediately after the burn trauma and is followed by the “Flow” Phase. In the first 2–4 days, the “Ebb” Phase develops. This phase partially overlaps with the “burn-shock” phase, which is characterized by a hypovolemic shock. It is a period with decreased metabolic rate, reduced cardiac output, hypovolemia [98], centralization of the circulation towards vital organs (brain and heart), and reduced tissue perfusion of the other organs, including the kidneys [99]. The kidneys are affected by hypoxia, and renal filtration rate is decreased [100].

Towards the 5th day, the “Flow” Phase is established [101]. It is a hyperdynamic, hypercatabolic state [101] in which plasma volume is increased, cardiac output augments, and perfusion of the kidney (and other organs) is improved [16,102], at least partially. The consequence is the development of AKI due to postburn RIRI (Renal Ischemia–Reperfusion Injury). This type of injury is also encountered after renal vascular occlusion, kidney transplantation, resuscitation after cardiac arrest [103].

During acute ischemia in the kidneys, prolyl-4-hydroxylases cannot perform the hydroxylation of proline residues (Pro402 and Pro564 in HIF-1α; Pro405 and Pro531 in HIF-2α), this process being iron-dependent and oxygen-dependent [104]. The un-hydroxylated HIF-α subunit cannot bind to pVHL as part of an E3-ubiquitin ligase complex, a complex that would undergo proteasomal degradation in normoxic conditions [69,104]. Consequently, HIF-α subunit degradation is inhibited [69,104]. The hypoxic stabilization of HIF-α is followed by its translocation into the cell nucleus, where it forms a heterodimer with HIF-β, that binds to the HRE domain of HIF target genes [104].

In renal epithelial cells, there are the three types of prolyl-hydroxylases—differentiated by specific PHD (prolyl-hydroxylases domain), i.e., PHD1, PHD2, PHD3 [105]—that manifest differences in activity level and expression in a manner that presents tissue specificity and cell specificity [106]. Different levels of hypoxia induce isoform-specific patterns of PHD in different cell types, which allows a flexible regulation of HIF in relation to oxygen levels [106]. PHD3 is expressed in the nucleus and cytoplasm, PHD2 is expressed in the cytoplasm, while PHD1 is expressed in the nucleus [107]. The renal tissue is not homogenously oxygenated; it was proved that kidney regions with lower oxygenation (such as the collecting ducts in the inner medulla, distal convoluted tubules, and the thick ascending limbs) have augmented levels of PHDs [107]. In normoxia, HIF-α is hydroxylated, especially by PHD2 [108]. In the process of reperfusion and reoxygenation, in the post-burn Flow Phase, HIF-α is preferentially hydroxylated by PHD3 [106].

In addition to PHDs that control the expression level of HIF-α, FIH (factor inhibiting HIF) is another oxygen-sensitive hydroxylase that regulates HIF transcription activity [109]. FIH has been identified in the kidney in Bowman capsule podocytes and in epithelial cells of the distal tubules [105,110]. When hypoxia increases beyond a certain level, FIH is inactivated [111] and cannot hydroxylate the asparaginyl residue of HIF-α (Asn803 in HIF-1α and Asn851 in HIF-2α). Consequently, the recruitment of CBP/p300 coactivators is permitted, which results in the augmentation of the transcriptional activity of HIF [109,112,113].

### 3.4. HIFs and Mitochondria in Patients with Major Burns

Mitochondria activities are severely disturbed in the presence of major burns. In fact, the burn literature refers to this as burn-related mitochondrial dysfunction [114,115]. Mitochondria-specific damage seems to appear very early as a response to burn injury, fragments of mitochondrial DNA being detected immediately after a burn [115]. As early as 15 min after a burn, cytochrome c is released from the mitochondria into the cytosol [101,114], and in 1 h, mitochondrial membrane potential changes occur [101,114]. Experimental studies on rats proved a decrease in the concentration of cytochrome a, b, and c in the kidney mitochondria by at least 70%, 8 h after the infliction of third-degree burns [116]. These studies noted a reduction of the phosphorylation activity in kidney mitochondria and a decrease in respiratory control ratio and state 3 respiration [116].

The relationship between mitochondria and HIF signaling pathway is complex and suggests the role of mitochondria as oxygen sensors [117]:

Mitochondrial complex III generates ROS [118,119] that change the redox state of enzyme-bound iron [120,121,122], resulting in the inhibition of the activity of PHDs, with consequent stabilization of HIF-α [120]. ROS production in the mitochondria is mainly the result of the activity of Electron Transport Chain (ETC) complexes I and III [123]. Less than 5% of ROS are generated by the activity of enzymes such as monoamine oxidase (MAO), cytochrome b5 reductase, nicotinamide adenine dinucleotide phosphate oxidase (Nox) [124]. During hypoxia, the ETC complex II becomes an important source of ROS, due to the change of the oxidation of NAD-related substrates to succinate oxidation [125]. In this situation, succinate acts as a signaling molecule involved in the transcription of HIF-1. This mechanism might partially explain why previous studies reported that in hypoxic conditions, ROS production remained constant (as in normoxia) or even increased [117,126].

### 3.5. HIFs and Reactive Oxygen Species/Reactive Nitrogen Species in the Presence of Major Burns

The stabilization of HIFs during hypoxia interferes with the production of ROS in two possible ways: by inhibiting the production of ROS [127] or by stimulating the production of ROS [128]. The hypoxic accumulation of HIF-1α upregulates the expression of Pyruvate Dehydrogenase Kinase isoform 1 (PDK1) [127,129]. Through phosphorylation of the pyruvate dehydrogenase subunits PDHA1 and PDHA2, PDK1 inhibits pyruvate dehydrogenase (PDH) activity, resulting in the inhibition of the oxidative decarboxylation of pyruvate and of the formation of acetyl-coenzyme A (acetyl-CoA) [127]. Therefore, a reduced quantity of acetyl-CoA enters the Krebs cycle. Finally, by decreasing mitochondrial oxygen consumption, ROS production is downregulated [127]. It was proved that HIF-1α stabilization increased the expression of microRNA-210 (miR-210) [130], which inhibits mitochondrial oxidation–reduction reactions via repression of Iron–Sulfur Cluster assembly proteins (ISCU1/2) [130]. Reduced mitochondrial respiration results in the decreased production of ROS. The rationale of downregulating ROS production through HIF accumulation is to protect the cells from apoptosis in response to hypoxia and oxidative stress [131]. Although it is generally agreed that HIFs stabilization in hypoxic conditions inhibits the mitochondrial production of ROS, there are studies that show positive feedback between HIFs accumulation and ROS production during hypoxia [128,132,133]. It appears that HIF-1α stabilization during hypoxia generates superoxide, with the consequent increased production of ROS. The mechanism involved would be the overexpression of the genes for NADPH Oxidase 1 (Nox1) and NADPH Oxidase 2 (Nox2) induced by HIF-1α [128].

NO is a reactive nitrogen species that decreases the ubiquitination of HIF-1α, alters the interactions between HIF-1α and pVHL, and inhibits HIF hydroxylation by PHDs [134,135]. These actions result in the accumulation of HIF during hypoxia. Other studies proved that NO induces HIF stabilization both in hypoxia and in normoxia, through mitochondria-dependent and -independent pathways [136].

## 4. Hypoxia, Inflammation, HIFs, and Kidney Lesions in Patients with Severe Burns

**Figure 2:**
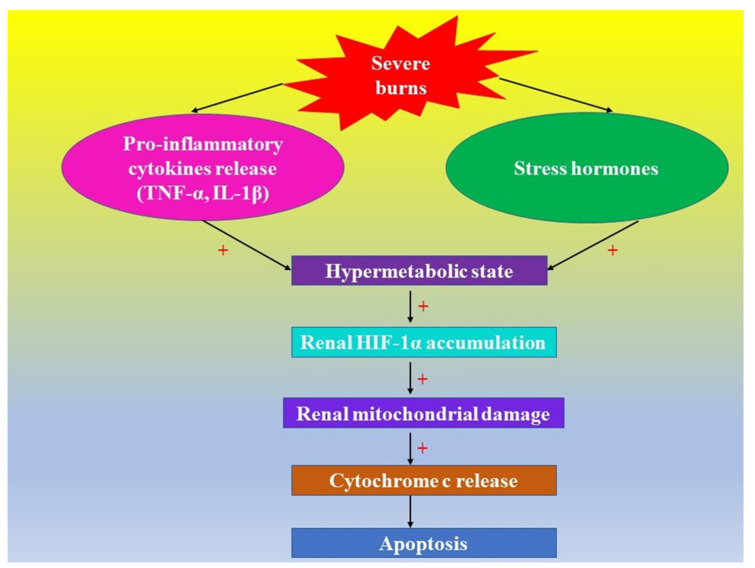
Severe burns and the effects of renal HIF-1α accumulation induced by a hypermetabolic state.

There is a strong connection between hypoxia, HIFs, inflammation, and kidney lesions in patients with burns. The systemic inflammatory response installs rapidly (after 4 h) in the presence of burns involving more than 30% of the TBSA [137,138]. In the presence of severe burns, there is an augmentation of the levels of the pro-inflammatory cytokines TNF-α and IL-1β, which increases the formation of ROS [139], resulting in HIF-1α stabilization [140,141]. The increased levels of cytokines are persistent in the “flow” phase of severe burns for about 6 weeks [138,142] and induce a systemic inflammatory response; they also contribute, together with stress hormones (Figure 2), to the development of a hypermetabolic state that might persist up to 36 months [137].

One of the main characteristics of the hypermetabolic state is persistent insulin resistance in peripheral tissues, including the kidneys [143]. It is interesting to note that HIF-1α (which is stabilized through multiple mechanisms in patients with severe burns) was reported to be a mediator of insulin resistance [144]. Hyperinsulinemia is one of the hallmarks of insulin resistance, and insulin was demonstrated to be an important stabilizer of HIF-1α both in hypoxia and in normoxia [101,145]; it seems that the activation of the PI3K/AKT pathway in hypoxic conditions determines HIF-1α accumulation [145,146] not through the inhibition of HIF prolyl-hydroxylation [69], but through the augmentation of HIF-α protein translation [147]. Therefore, it appears that hypoxia and HIF-α accumulation promotes tissue inflammation and accentuates insulin resistance [144,148]. Hypoxia induces the stabilization of HIF-1α, which has a binding site on the CD18 gene, which encodes the common subunit of the four types of beta2 integrin heterodimer [149]. Beta2 integrin levels increase in hypoxia, and this molecule is essential for the adhesion of leukocytes to the activated endothelium [149]; these aspects prove that leukocyte adhesion during hypoxia is mediated by HIF-1-dependent induction of beta2 integrin gene expression [149]. It is common knowledge that HIF-1α is essential for the regulation of glycolytic activity in the cells, including granulocytes and monocytes/macrophages controlling optimal ATP production [150]. Experimental studies in mice revealed that when HIF-1α activity is absent, the cellular ATP levels drop, resulting in the impairment of motility, invasiveness, aggregation, and bactericidal action of myeloid cells; this proves that HIF-1α is necessary for myeloid cell-mediated inflammation [151].

HIF-1α accumulated in hypoxia modulates the development and functions of lymphocytes B and lymphocytes T and regulates T cell receptor signal transduction [152,153,154]. One can observe there is reciprocal positive feedback between inflammatory cytokines in burns and HIF-1 accumulation and actions in hypoxic renal tissue.

## 5. HIFs and Acute Hypoxic Cell Death in Kidneys in Severe Burns

As already discussed, in hypoxic conditions, PHD hydrolytic action is inhibited. Consequently, HIF-α accumulates, enters the nucleus, and dimerizes with HIF-β, forming HIF [69,91]. Depending on the cell type and on the cellular environment, HIF induces the expression of genes involved in cellular metabolism, cell proliferation, angiogenesis, extracellular matrix formation, and apoptosis [109]. HIF controls mechanisms and biological processes involved in cell survival in hypoxic conditions, such as protein translation, mitochondrial signaling, anaerobic glycolysis, hypoxic cell death [109]. The inductive or protective effect of HIF upon apoptosis in hypoxia depends on the cell type and the cellular context [155,156].

In hypoxic renal tissue, HIF-1α was detected in the epithelial cells of the renal tubules, while HIF-2α was found in endothelial cells and interstitial kidney fibroblasts [157], pleading for HIFs’ different roles in different cells [158,159]. In certain situations, HIF plays a pro-apoptotic role [155], in other situations it has a protective role against hypoxic apoptosis, depending on glucose availability in the cell [155,160]. There is a complex interaction between the electron transport chain (complexes I, II, III) and HIF-α [122,130]. It is also known that, at the level of cytochrome c, the electron transport chain processes and the apoptosis process overlap [161]: under the action of apoptotic stimuli, cytochrome c is released into the cytosol, from the mitochondrial intermembrane space, and triggers programmed cell death (Figure 2) [161]. This is a described phenomenon in severe burns [114,139]. It was also proven that HIF-α can regulate some proteins involved in apoptosis, such as the members of the Bcl-2 family (B-cell lymphoma protein-2) [162], which are proved to control cytochrome-c-mediated apoptosis [161]. Consequently, one can affirm the existence of a crosstalk between the HIF-α signaling pathway and the apoptotic signaling pathway, at the mitochondrial level and in the cytosol [163,164] in many situations characterized by hypoxia, including severe burns [101].

HIF-α might have anti-apoptotic effects through (i) the induction of Bcl-xL (B-cell lymphoma-extra-large), which is a mitochondrial transmembrane molecule, belonging to the Bcl-2 family of proteins. It prevents the release of cytochrome c from the mitochondria into the cytosol and has an anti-apoptotic effect [165,166]; (ii) the induction of Mcl-1 (Myeloid cell leukemia 1) [167,168], which is a member of the Bcl-2 family of proteins, with anti-apoptotic action [169,170]. It inhibits the permeabilization of the mitochondrial outer membrane and the release of cytochrome C from the mitochondria into the cytosol [171,172]; (iii) the decrease of the levels of Bax, Bak, and Bid [173,174], which are pro-apoptotic proteins, members of the Bcl-2 family [175,176]. Bax and Bak accumulate in the mitochondrial outer membrane (under the influence of apoptotic stimuli); here, they oligomerize and contribute to the permeabilization of the mitochondrial outer membrane, followed by the release of cytochrome c from the mitochondria [177,178]. Under the action of multiple proteases, Bid is activated to tBid (truncated Bid), which is translocated from the cytosol into the mitochondrial membrane [179,180]; here, it acts directly and in cooperation with Bax and Bak, resulting in the release of cytochrome c from the mitochondria into the cytosol [179,180,181]; (iv) the induction of Bcl-2 family proteins with an anti-apoptotic role [173].

In a different cellular context, HIF-α has pro-apoptotic effect through: (i) the induction of Noxa [182]; Noxa is a Bcl-2 homology 3 (BH3)-only member of the Bcl-2 family of proteins [183]; it undergoes BH3 motif-dependent localization to the mitochondria, where it interacts with other members of the Bcl-2 family (such as Mcl-1) [184], with consequent activation of Caspase-9 [185]. Activated caspase-9, within the apoptosome, promotes the activation of downstream caspases 7, 6, and 3 [185]; (ii) the induction of BNIP3 (Bcl2/adenovirus EIB 19kD-interacting protein 3) [186,187,188]; accumulation of BNIP3 protein induces the classical intrinsic apoptosis pathway, through cytochrome c and caspase complex activation, or may trigger autophagic cell death, without cytochrome c involvement [189,190]; (iii) the induction of Nip-3-like protein 3 [186,187] that is a homologous of BNIP3, also called Bcl-2-interacting protein 3-like; it binds to Bcl-2 and determines, at the mitochondrial level, the loss of membrane potential and the release of cytochrome c in the cytosol [191,192]; (iv) the downregulation of Bcl-2 family proteins with a pro-apoptotic role [173,193,194].

The Bcl-2 family proteins, that are regulated among other factors by HIF-α, influence mitochondrial dynamics and are involved in ischemia-induced acute kidney injury [194,195].

Table 1 summarizes the complex roles played by HIFs in renal hypoxia in the context of burns.

## 6. Conclusions

In the presence of severe burns, one of the consequences of the post-combustion shock is represented by the centralization of the circulation with hypoxia in all tissues, excepting the heart and the brain. Renal hypoxia is the main cause of AKI and acute renal failure in patients with burns exceeding 20–30% of TBSA. This narrative review suggests that HIFs are the key factors that interconnect hypoxia, systemic inflammatory response, apoptosis, and kidney lesions in patients with severe burns. Depending on the cell environment, in some situations, HIFs are proapoptotic factors while in other situations, they are antiapoptotic factors. The modulation of HIFs might prevent the development of kidney lesions in hypoxic conditions, including in the presence of severe burns. Further studies are necessary to describe the effects of well-known HIFs modulators upon AKI emergence in patients with severe burns.

## Figures and Tables

**Table 1:** The complex roles played by HIFs in renal hypoxia in the context of burns.

	Research Subject	References
1.	Acute kidney injury—activator of HIFs	[69,70]
2.	HIF signaling pathway might be activated by: - TNF-α - IL-1β - insulin - insulin-like growth factors - angiotensin II - nitric oxide - ROS	
		[71]
		[72,73]
		[73,74]
		[74,75,76]
		[77]
		[78,79,80]
		[81,82,83]
3.	As transcriptional factors, HIFs regulate the expression of genes involved in oxygen delivery to the renal tissues, triggering adaptation to hypoxia in the kidney	[69,70]
4.	HIFs are upregulators of the genes encoding most of the glycolytic enzymes	[86,88]
5.	HIF target genes (hypoxia-sensitive genes) induce the synthesis of EPO, VEGF, PGK-1, GLUT-1, transferrin and transferrin receptor, enolase 1, LDH-A (lactate dehydrogenase A), CTGF (connective tissue growth factor), vital for kidney functionality in normal conditions and for kidney adaptation to hypoxia	[15,70,91]
6.	Hypoxic stabilization of HIF-α	[104]
7.	In normoxia, HIF-α is hydroxylated, especially by PHD2In the process of reperfusion and reoxygenation, in the post-burn Flow Phase, HIF-α is preferentially hydroxylated by PHD3	[106,108,109]
8.	FIH (factor inhibiting HIF)—another oxygen-sensitive hydroxylase that regulates HIF transcription activity	[105,110,111,112,113]
9.	Mitochondria and HIF signaling pathway complex relationship	[117]
10.	HIFs stabilization in hypoxia interferes with ROS generation in two ways: - by inhibiting the production of ROS - by stimulating the production of ROS	[127,128,129,131,132,133]
11.	HIF-1α stabilization increases the expression of miR-210 (microRNA-210)	[130]
12	NO (nitric oxide) and HIFs relationship during hypoxia	[134,135,136]
13.	In severe burns, the pro-inflammatory cytokines TNF-α and IL-1β increase ROS formation, triggering HIF-1α stabilization	[139,140,141]
14.	HIFs and insulin resistance	[139,144,145,146,148]
15.	In hypoxic renal tissue: - HIF-1α was detected in the epithelial cells of the renal tubules. - HIF-2α was found in endothelial cells and interstitial kidney fibroblasts.	[157,158,159]
16.	HIF-α and apoptosis in burns	[101,161,162,163,164]
17.	Anti-apoptotic effects of HIFs through: - induction of Bcl-xL (B-cell lymphoma-extra-large) - induction of Mcl-1 (Myeloid cell leukemia 1) - decrease of the levels of Bax, Bak, and Bid, pro-apoptotic proteins, members of the Bcl-2 family - induction of Bcl-2 family proteins with an anti-apoptotic role	
		[165,166]
		[167,168,169,170,171,172]
		[173,174,175,176,177,178,179,180,181]
		[173]
18.	Pro-apoptotic effects of HIFs through: - induction of Noxa - induction of BNIP3 (Bcl2/adenovirus EIB 19kD-interacting protein 3) - induction of Nip-3-like protein 3it is a homologous of BNIP3, also called Bcl-2-interacting protein 3-like; - downregulation of Bcl-2 family proteins with a pro-apoptotic role	
		[182,183,184,185]
		[186,187,188,189,190]
		[186,187,188,191,192]
		[173,193,194,195]

## Data Availability

Not applicable.
